# Supplementary material for: Differential Modulation of Cerebellar Flocculus Unipolar Brush Cells during Vestibular Compensation
Source: Biomedicines. 2023 Apr 27;11(5):1298. doi: 10.3390/biomedicines11051298 (PMC10215535; doi:10.3390/biomedicines11051298)
Supplement: Supplementary file 1 [file biomedicines-11-01298-s001.zip › biomedicines-2247954-supplementary.pdf]

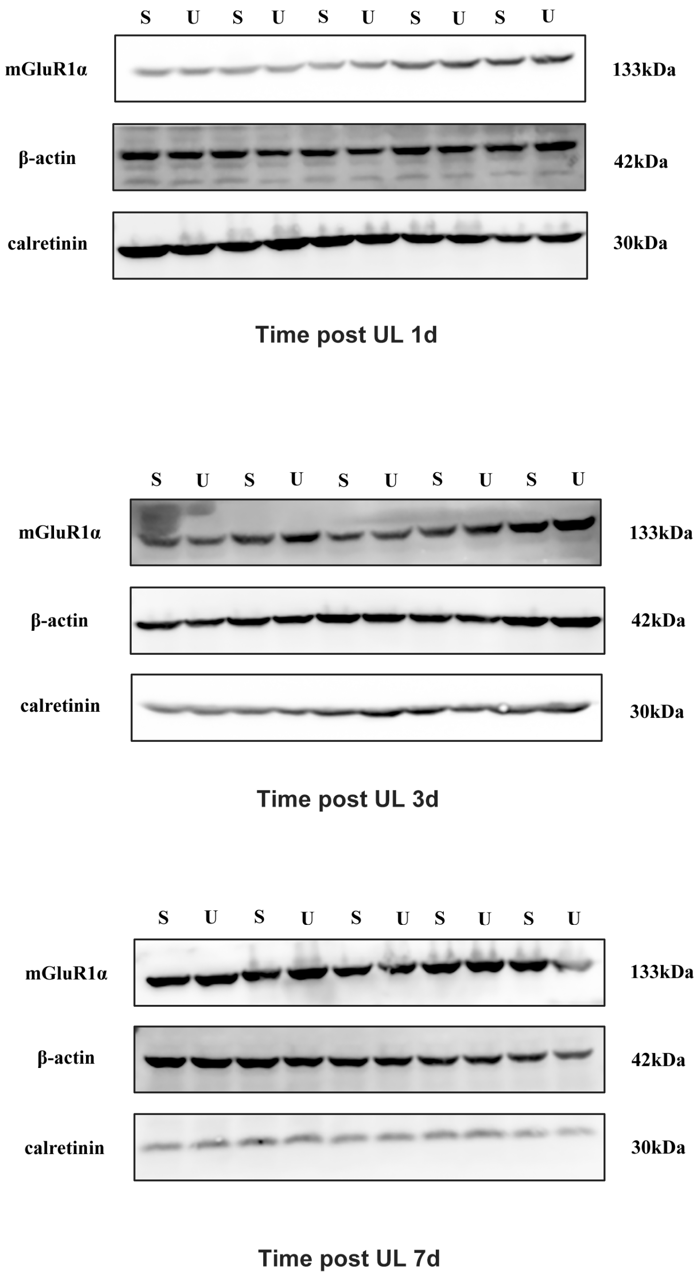

Figure S1. mGluR1α and calretinin expression in the flocculus 1d, 3d and 7d after UL. Western blot for mGluR1α and calretinin in the ipsilateral flocculus at the 1d, 3d, and 7d following UL compared to sham controls. Six micrograms of protein were applied in each lane (n = 5 for both UL and sham groups).
